# Subacute Haemorrhage in the Seminal Vesicle as a Cause of Persistent Haematospermia: A Case Report

**DOI:** 10.7759/cureus.45709

**Published:** 2023-09-21

**Authors:** Abdallah Sharqawi, Sarah Mostafa

**Affiliations:** 1 Urology, Faculty of Medicine, Suez Canal University, Ismailia, EGY; 2 Neuropsychiatry, Faculty of Medicine, Suez Canal University, Ismailia, EGY

**Keywords:** semen, subacute haemorrhage, mri pelvis, haematospermia, seminal vesicle bleed

## Abstract

Haematospermia is a relatively uncommon condition that can be caused by a variety of factors, including infection, inflammation, trauma, and neoplastic disease. Bleeding from the seminal vesicle is considered to be a rare cause. We present a case of a 65-year-old man who had repeated episodes of haematospermia over the previous few years. Initial physical examination was unremarkable. Laboratory tests, including coagulation profile and prostate-specific antigen level, were within normal limits. A magnetic resonance imaging scan of his pelvis revealed a right seminal vesicle haemorrhage as the cause of his haematospermia. The patient was reassured and was managed conservatively.

## Introduction

Haematospermia, which is the presence of blood in semen, can cause distress and anxiety in sexually active males and their partners. It is a relatively rare urological symptom, accounting for about 1% of cases [1]. The most common causes of haematospermia are trauma and infections, but it can also be caused by malignancy or other rare conditions such as severe hypertension, bleeding disorders, or liver cirrhosis. Often, no specific cause can be identified, so it is important to reassure patients and ease their concerns. MRI of the pelvis can aid in identifying the origin of bleeding from the seminal vesicles and rule out potentially serious causes of haematospermia [2].

## Case presentation

**Figure 1:**
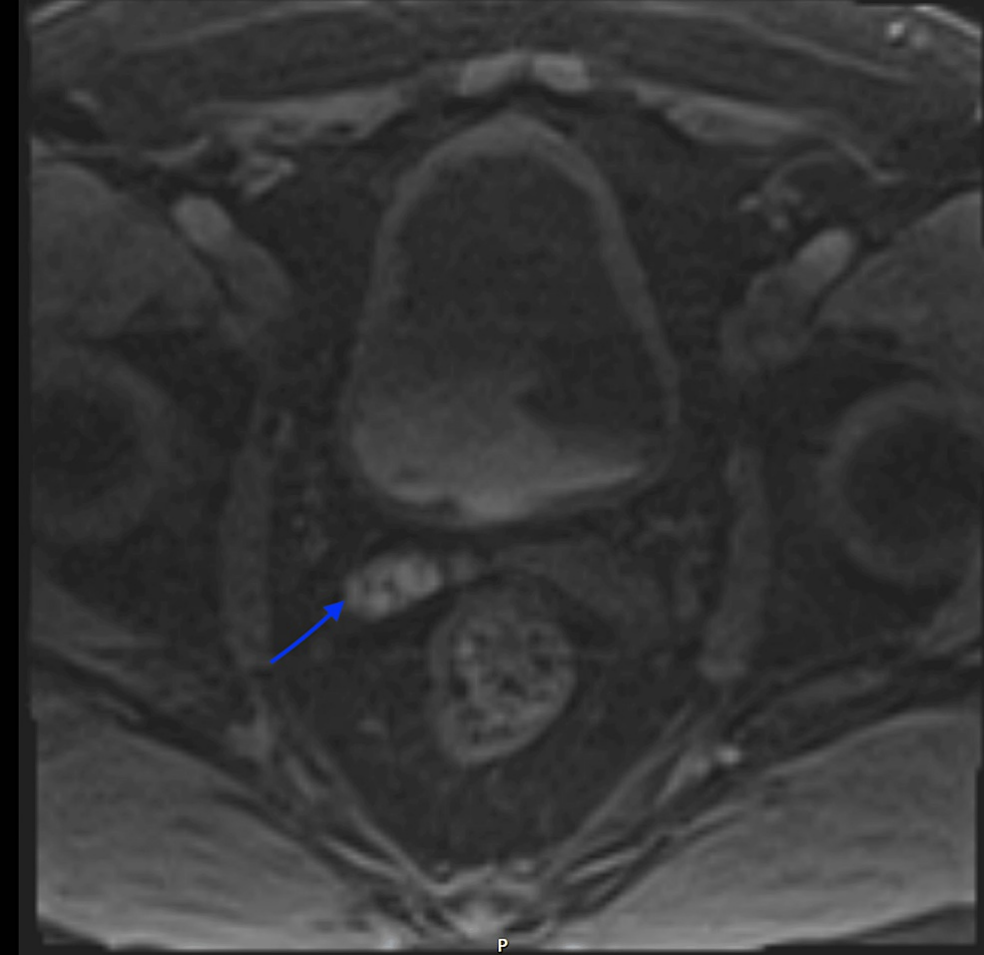
T1-weighted MRI showing high signal intensity in the right seminal vesicle (arrow)

**Figure 2:**
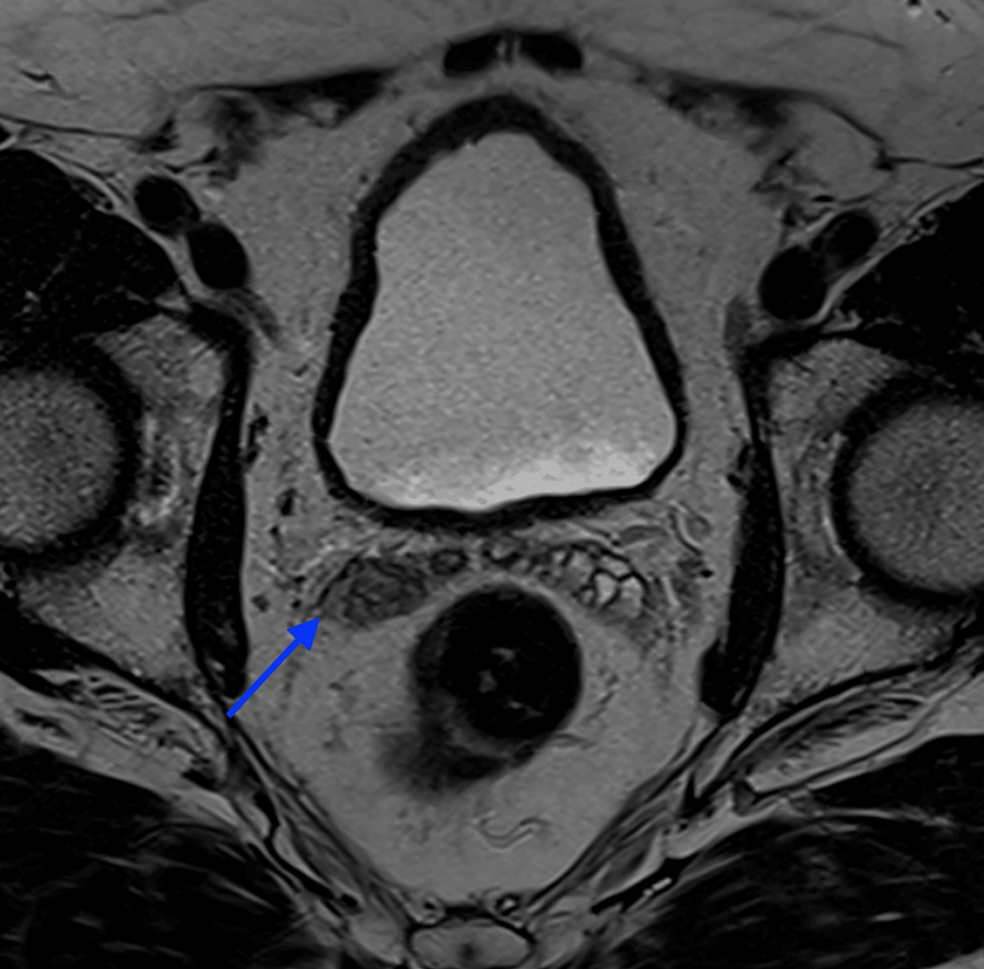
T2-weighted MRI showing low signal intensity in the right seminal vesicle (arrow)

A 65-year-old man with no previous medical conditions and not taking anticoagulants presented to the urology clinic at Suez Canal University Hospitals with intermittent episodes of self-resolving haematospermia over several years. When the patient presented with daily persistent haematospermia for a week, he was referred on a two-week wait pathway. A detailed examination, including a digital rectal exam of the prostate, found no noteworthy issues, and bleeding from his female partner was eliminated as a possible cause. Prostate-specific antigen (PSA) blood test, urine culture, semen culture, and flexible cystoscopy were all performed, but no abnormalities were detected. As the patient continued to experience haematospermia even after masturbation, a pelvic MRI was performed to rule out any hidden intrapelvic conditions. The MRI revealed a subacute haemorrhage in the right seminal vesicle, with a high signal intensity on the T1-weighted sequence and a low signal intensity on the T2-weighted sequence. No focal enhancing lesion was found, and it was determined that this was the cause of the haematospermia (Figures 1, 2). The patient was advised to abstain from ejaculation for a few months and was reassured. Three months later, no similar episodes were reported, and the patient has been monitored for a year without any recurrence.

## Discussion

Haematospermia commonly affects young males and is usually a benign and self-limited symptom that can be managed with basic investigations and simple treatments. However, in some cases, it may indicate urogenital malignancy, particularly in patients over 40 years of age or those with persistent or recurrent symptoms. Therefore, further investigations are required. Imaging and endoscopic examinations are necessary to make a definitive diagnosis and provide appropriate treatment. MRI of the pelvis is an excellent diagnostic tool for investigating the underlying cause of haematospermia [3].

In this case, the MRI findings revealed subacute haemorrhage in the right seminal vesicle, with high signal intensity in T1-weighted sequence and low signal intensity in T2-weighted sequence, which is influenced by the magnetic properties of iron and the integrity of the red blood cell membrane. Iron is diamagnetic when oxygenated and paramagnetic when deoxygenated, and in its paramagnetic state, iron atoms shorten the T1 and T2 relaxation times, with a greater effect on T1-weighted images [4]. The evaluation of haemorrhage and these MRI features is essential when interpreting imaging results to differentiate between different stages of haemorrhage based on signal changes in MRI. Acute haemorrhages appear iso- or hypointense on T1-weighted MRI and hypointense on T2-weighted MRI. Early subacute haemorrhages appear hyperintense on T1-weighted MRI and hypointense on T2-weighted MRI. Late subacute haemorrhages appear hyperintense on both T1-weighted and T2-weighted MRI, with a rim of low intensity. Chronic haemorrhages appear hypointense on both T1-weighted and T2-weighted MRI [5].

The bleeding from the seminal vesicles can be caused by various factors, such as trauma, infection, inflammation, or tumours. Inflammation or infection of the seminal vesicles is known as vasculitis, which may result in pain in the groin, fever, and blood in semen. Studies have shown that vasculitis is an infrequent cause of haematospermia, accounting for only 2% to 5% of cases [6]. Tumours or growths in the seminal vesicles may also lead to haematospermia. These growths can be benign or malignant and may necessitate further examination or surgical treatment. According to previous research, tumours of the seminal vesicles are uncommon, accounting for only 1% to 2% of all genitourinary tract tumours [7]. The diagnosis of haematospermia due to seminal vesicle bleeding involves physical examination, medical history, and laboratory tests, along with imaging studies such as ultrasound or MRI to detect any abnormalities. Treatment depends on the underlying cause, with antibiotics prescribed for bacterial infections and surgery necessary for tumours or growths. Most cases are treated conservatively with antibiotics or anti-inflammatory medications [8].

The potential for urological malignancy to cause haematospermia, especially in patients over 40 years of age, was also highlighted in studies by Zhao et al. and Suh et al., which found cases of prostate cancer, seminal vesicle cancer, and bladder cancer among patients with haematospermia, as well as common calcifications in the prostate, seminal vesicles, and ejaculatory ducts [9,10]. These findings suggest that additional investigations, such as transrectal MRI and prostate-specific antigen testing, should be considered to rule out urological malignancy in patients with haematospermia.

## Conclusions

Seminal vesicle bleeding is a rare cause of haematospermia, with vasculitis and tumours being the most common causes. MRI is a useful diagnostic tool for determining the underlying cause of haematospermia, and treatment depends on the cause. In most cases, conservative treatment is sufficient and includes antibiotics or anti-inflammatory medications. It is also important to consider the possibility of the female partner as the source of bleeding in sexually active patients and rule it out.
